# Effect of an mHealth weight loss intervention on Healthy Eating Index diet quality: the SMARTER randomised controlled trial

**DOI:** 10.1017/S0007114523001137

**Published:** 2023-06-07

**Authors:** Jessica Cheng, Tina Costacou, Susan M. Sereika, Molly B. Conroy, Bambang Parmanto, Bonny Rockette-Wagner, Andrea M. Kriska, Mary Lou Klem, Lora E. Burke

**Affiliations:** 1 Department of Epidemiology, School of Public Health, University of Pittsburgh, Pittsburgh, PA, USA; 2 Department of Health and Community Systems, School of Nursing, University of Pittsburgh, Pittsburgh, PA, USA; 3 Department of Internal Medicine, School of Medicine, University of Utah, Salt Lake City, UT, USA; 4 Department of Health Information Management, School of Rehabilitation Sciences, University of Pittsburgh, Pittsburgh, PA, USA; 5 Health Sciences Library System, University of Pittsburgh, Pittsburgh, PA, USA

**Keywords:** Diet quality, Mobile health, Weight loss, Behaviour change

## Abstract

In the few weight loss studies assessing diet quality, improvements have been minimal and recommended calculation methods have not been used. This secondary analysis of a parallel group randomised trial (regsitered: https://clinicaltrials.gov/ct2/show/NCT03367936) assessed whether self-monitoring with feedback (SM + FB) *v*. self-monitoring alone (SM) improved diet quality. Adults with overweight/obesity (randomised: SM *n* 251, SM + FB *n* 251; analysed SM *n* 170, SM + FB *n* 186) self-monitored diet, physical activity and weight. Real-time, personalised feedback, delivered via a study-specific app up to three times daily, was based on reported energy, fat and added sugar intake. Healthy Eating Index 2015 (HEI-2015) scores were calculated from 24-hour recalls. Higher scores represent better diet quality. Data were collected August 2018 to March 2021 and analysed spring 2022. The sample was mostly female (78·9 %) and white (85·4 %). At baseline, HEI-2015 total scores and bootstrapped 95 % CI were similar by treatment group (SM + FB: 63·11 (60·41, 65·24); SM: 61·02 (58·72, 62·81)) with similar minimal improvement observed at 6 months (SM + FB: 65·42 (63·30, 67·20); SM: 63·19 (61·22, 64·97)) and 12 months (SM + FB: 63·94 (61·40, 66·29); SM: 63·56 (60·81, 65·42)). Among those who lost ≥ 5 % of baseline weight, HEI-2015 scores improved (baseline: 62·00 (58·94, 64·12); 6 months: 68·02 (65·41, 71·23); 12 months: 65·93 (63·40, 68·61)). There was no effect of the intervention on diet quality change. Clinically meaningful weight loss was related to diet quality improvement. Feedback may need to incorporate more targeted nutritional content.

Overweight and obesity rates in the USA and across the world are high and continue to rise^(1,2)^. As such, extensive research has been conducted on developing and testing behavioural interventions for weight loss. Diet and exercise are recognised as key components of standard behavioural weight loss programmes with the US Preventative Services Task Force recommending such multicomponent interventions for adults with obesity^(3)^. However, many studies explicitly focus goals on energy restriction and low/moderate fat intake. While nutritional counselling in interventions may discuss other aspects of diet, such as increasing fruit intake, it is often couched in terms of helping with the energy and fat restriction goals. Few interventions report on dietary changes holistically; therefore, it is unclear if and how weight loss interventions affect diet quality.

Diet quality represents the healthfulness of an individual’s overall dietary pattern. Poor diet quality is a major correlate of prevalent overweight and obesity^(4)^ as well as weight gain over time^(5,6)^. In randomised controlled trials, improvements in diet quality have been shown to relate to weight loss separate from the effect of energy restriction^(7,8)^. Diet quality has also been shown to relate to weight loss maintenance post-intervention^(9,10)^.

Importantly, diet quality measured using validated instruments (e.g. dietary recalls and food frequency questionnaires) and multiple indices (i.e. Healthy Eating Index, Alternate Healthy Eating Index, alternate Mediterranean Diet score and adherence to the Dietary Approaches to Stop Hypertension diet) has also been shown to relate to cardiometabolic risk factors other than weight^(4,11)^ and to chronic disease independent of its effect on weight^(12)^. Indeed, women with obesity who have better diet quality are more metabolically healthy than those with poor diet quality^(13)^. Since weight loss can be difficult to achieve and weight regain is common^(14)^, diet quality change may provide an important additional endpoint for assessing the effectiveness of interventions for chronic disease prevention.

The provision of personalised dietary feedback may be particularly important for diet quality improvement. In a small pilot study among participants self-monitoring their diet using an app-based food diary (i.e. tracking foods as they were eaten throughout the day), who were not provided dietary feedback, diet quality worsened over 8 weeks. This was in comparison to participants using the Notepad app on the phone or paper-and-pencil methods of self-monitoring who did receive feedback and who improved diet quality^(15)^.

This secondary analysis expands on the potential importance of providing dietary feedback to adults with overweight or obesity that was suggested by the results of the pilot study. In a larger sample and over 12 months, we assessed the association between diet quality and a mHealth intervention of self-monitoring (via Fitbit food diary) and personalised, automated feedback compared with self-monitoring alone. We hypothesised that the provision of feedback would result in improved diet quality over self-monitoring alone. We also assessed the relationship between diet quality improvement and weight loss with the hypothesis being that we would observe greater diet quality improvement among those with clinically meaningful weight loss compared with those without. In addition to establishing the importance of dietary feedback in a remote, scalable intervention, such an examination might help identify how the intervention can be refined for maximal benefit.

## Methods

### Study sample

The design of SMARTER, a 12-month parallel group randomised controlled trial, as well as the primary outcome of weight loss have been described elsewhere^(16–18)^ (online Supplementary Fig. S1). Briefly, using randomisation software staff randomised adults with overweight or obesity with equal allocation stratified by race and gender to either a group which received individualised feedback messages based on self-monitoring data (SM + FB) or to a self-monitoring only comparator (SM). Both groups were given energy, fat gram and physical activity goals. At baseline, all participants attended a one-on-one, 90-minute dietary counselling session with a master’s level registered dietitian who had prior experience in standard behavioural treatment. Both groups were instructed to weigh themselves daily on a study-provided digital scale, record all foods and drinks in the Fitbit food diary and wear a Fitbit activity tracker to monitor physical activity. The University of Pittsburgh institutional review board approved the protocol. Participants were recruited from the greater Pittsburgh, Pennsylvania area (August 2018–March 2020), and participants provided written informed consent. Neither assessors nor participants were blinded to treatment assignment due to the behavioural nature of the intervention. This analysis was conducted in Spring 2022.

Dietary feedback messages were delivered to a study-specific smartphone app up to three times daily while physical activity and weight messages were sent 3–4 times per week and once a week, respectively. Although there was no specific diet quality goal, suggestions to eat better were inherent in the feedback messages the intervention group received (e.g. ‘A balanced breakfast includes different food groups, for example, whole grain toast, nut butter, and fruit.’). The type of message participants received was determined by algorithm conditions described previously^(16)^. Briefly, to select appropriate messages the algorithm used the energy, fat and added sugar intake reported in the Fitbit food diary along with consideration of the prescribed energy and fat goals. For example, if a participant recorded foods with fat content >30 % of the fat goal at breakfast, a message suggesting they limit fat for the remainder of the day may have been sent (e.g. ‘Choose lower fat foods as the day goes on to stay on track with your calorie and fat goal’).

Most of the over 2000 unique dietary feedback messages in the message library focussed on energy, fat and added sugar intake, as well as the importance of self-monitoring foods. Supplementary Table S1 provides examples of messages that address each component of the Healthy Eating Index-2015 (HEI-2015). The HEI-2015 aligns with the Dietary Guidelines for Americans (DGA) 2015–2020 and was used as the measure of diet quality in this study^(19)^. Importantly, while some components were never addressed, such as sodium (Na), there were multiple possible messages addressing other components (e.g. the word ‘fruit’ appears in over 100 unique messages).

### Measures

Demographic characteristics (e.g. race and ethnicity, income, education) and some clinical characteristics (i.e. blood pressure, high cholesterol, high trigylcerides [TAG] and smoking status) were self-reported at baseline. Height, weight, waist circumference and blood pressure were measured by trained staff at baseline and follow-up and BMI (kg/m^2^) was calculated based on weight and height measurements. At-home weight data from the WIFI-enabled scale was used in lieu of staff-collected data after the start of the COVID-19 pandemic in the USA (March 2020).

Dietary data were collected at baseline, 6 and 12 months on two separate days (i.e. up to 6 recalls total per participant throughout the study period) to minimise within-person random error. The 6-month time point was selected because other weight loss studies have shown improvement through 6 months and then weight regain. Dietary intake was assessed at the 12-month time point as it was the end of the intervention period. Dietary recalls were collected using the Automated Self-Administered 24-hour recall (ASA-24) system managed by the National Cancer Institute^(20)^. ASA-24 performs well compared with interviewer-administered recall^(21)^ with computer prompts imitating the multi-pass method. To minimise the potential for issues with usability, participants completed their first dietary recall while staff members trained in dietary recalls were present to answer questions. For subsequent recalls, which could be completed remotely, participants could reach out to staff for assistance.

HEI-2015 scores were calculated from dietary recalls. The HEI-2015 has demonstrated construct validity, reliability and criterion validity^(22)^ and is consistent with other dietary indices^(23)^. The HEI-2015 includes nine adequacy and four moderation components. For most components, intake per 1000 calories (1000 kcal = 4184 kJ) is scored. Values between the minimum and maximum score are scored proportionally. Component scores are summed to create an HEI total score with 100 being the best possible score^(19)^. Because the HEI is density-based, the correlation between HEI diet quality and diet quantity is low, suggesting a desirable independence between measures^(22)^. The average HEI-2015 score in the general population is 59^(24)^, well below a score of 74 which would satisfy Healthy People 2020 objectives^(25)^.

There are various methods of HEI calculation. As the bivariate^(26)^ and multivariate^(27)^ methods require sufficiently large datasets, these methods were not viable for use in our sample. Therefore, HEI-2015 total and component scores were calculated using the population ratio method as it is recommended for the assessment of intervention effects^(28)^. The population ratio method provides a less biased estimate of a population’s mean HEI score than the simple scoring algorithm or mean ratio method^(29)^.

### Statistical analysis

The distributions of continuous variables were assessed for normality using histograms and normal probability plots. For continuous-type normally distributed variables, comparison of baseline characteristics between those with complete data at all time points and those with missing data at 6 and/or 12 months was conducted using pooled variance *t* tests when group variances were equal (i.e. male waist circumference, systolic and diastolic blood pressure, education) or separate-variance *t* tests with the Satterthwaite method approximation of degrees of freedom when group variances were not equal (i.e. female waist circumference). Wilcoxon rank-sum tests were applied to compare group-specific distributions when the distribution of continuous-type variables was not normal (i.e. age, BMI and body fat percentage). Chi-square tests were used to compare distributions of categorical variables (i.e. sex, race and ethnicity, employment, household income, self-reported high blood pressure, high cholesterol and high TAG, smoking status) between those with and without missing dietary data. All analyses were performed using SAS version 9.4 (SAS Institute, Inc.).

The population ratio method was used to calculate the mean intake of dietary constituents across all individuals prior to constructing ratios and scoring the HEI-2015. Bootstrap resampling was utilised to estimate 95 % CI for mean HEI-2015 total and subcomponent scores for each study time point by treatment group and by clinically meaningful weight loss status with 200 bootstrap resamples generated. To test whether there were differences in HEI 2015 total scores at each timepoint by study arm and weight loss categorisation, CI were compared for overlap. As recommended, radar plots were used to visualise component scores of the HEI-2015^(19,30)^.

As there was no observed difference in mean weight loss, the primary outcome of the parent study, between study arms at either 6 or 12 months^(17,18)^; the study arms were combined when assessing the diet quality over time of (1) those achieving clinically meaningful weight loss (≥ 5 % of baseline weight) at the 6-month time point compared with those without clinically meaningful weight loss at the 6-month time point and (2) those achieving clinically meaningful weight loss at the 12-month time point compared with those without clinically meaningful weight loss at the 12-month time point.

A sensitivity analysis was performed comparing those participants in the SM + FB group who viewed less than the median percentage of FB messages over 12 months compared with those who viewed greater than or equal to the median percentage of FB messages. The percentage of FB messages viewed was calculated as the number of messages viewed divided by the number of intended messages. For participants completing the entire study, the number of messages they were supposed to receive was 1095 messages (i.e., 3 messages a day × 365 days).

## Results

Of the 502 participants enrolled (SM *n* 251; SM + FB *n* 251), 356 were included in this secondary data analysis as retention at 12 months was 78·5 %^(18)^ and because some participants retained in the study did not contribute dietary data at follow-up. Participants in this complete case analyses (Table 1) were mostly female (78·9 %), white (85·4 %), middle-aged (median = 51·0 years) and had obesity (median BMI = 33·1). Participants with complete dietary data (*n* 356) who were included in this analysis were defined as having ≥ 1 dietary recall at all timepoints. Analysed participants were significantly older (*P* < 0·0001), more likely to identify as white (*P* = 0·01), more likely to report high blood pressure (*P* < 0·01), and high cholesterol (*P* < 0·01) and had higher systolic blood pressure (*P* = 0·04) and lower BMI (*P* = 0·03) compared with those who were missing dietary data at 6 and/or 12 months. The percentage of participants with complete dietary data did not differ by treatment assignment (SM = 67·7% *v*. SM + FB = 74·1 %, chi-square *P* = 0·12).

**fig-1.**
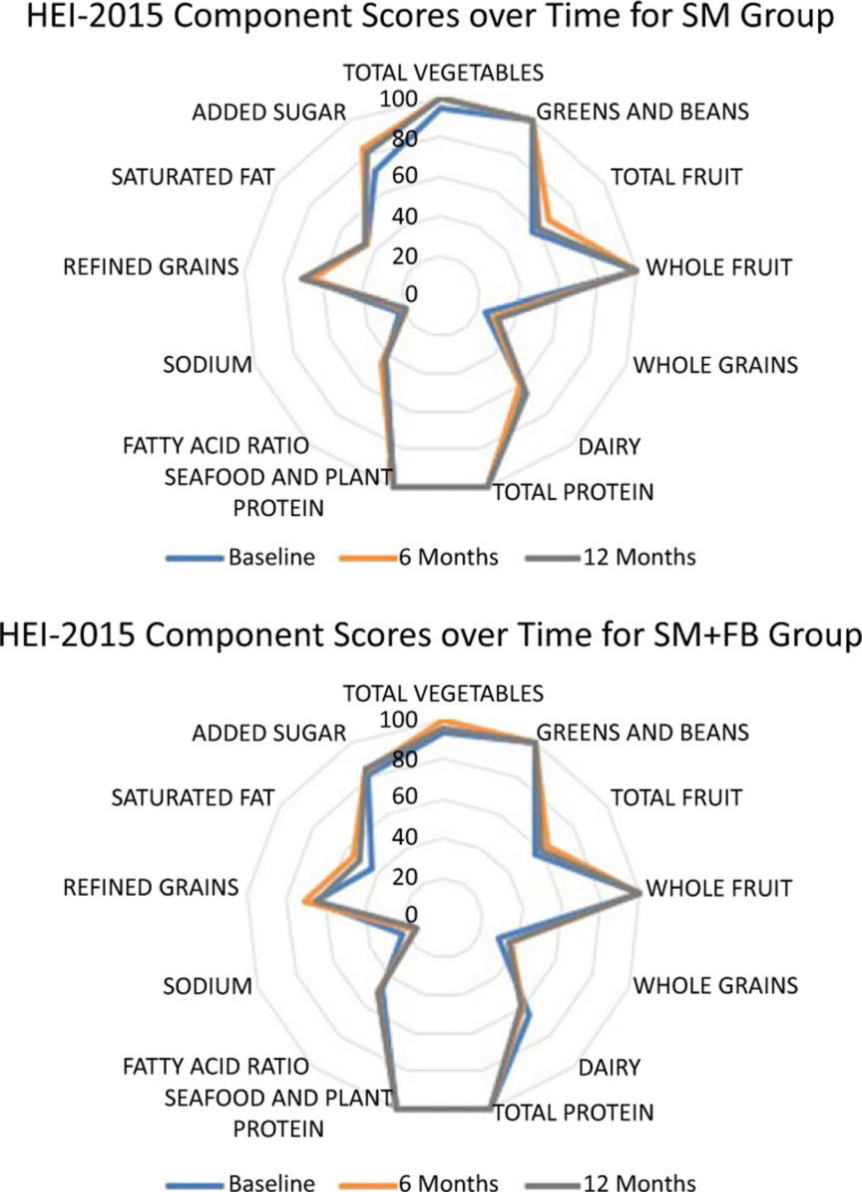
Radar Plot Depicting Component Scores of the Healthy Eating Index 2015 by Group at Each Time Point. Note: Scores touching the outer ring represent the maximum score for a component (100 % of the maximum score). A perfect diet quality score of 100 would be represented by touching the outer ring for all components.

At baseline, mean HEI-2015 total scores and bootstrapped 95 % CI were similar by treatment group (SM + FB: 63·11 (60·41, 65·24) *v*. SM: 61·02 (58·72, 62·81)) (Table 2). For both groups, little improvement in HEI-2015 total scores from baseline was observed at 6 months (SM + FB: 65·42 (63·30, 67·20) *v*. SM: 63·19 (61·22, 64·97)) or 12 months (SM + FB: 63·94 (61·40, 66·29) *v*. SM: 63·56 (60·81, 65·42)). Similarly, changes in component scores were small between groups and across time points. Figure 1 depicts component scores at each time point by treatment group.

**Fig. 2.**
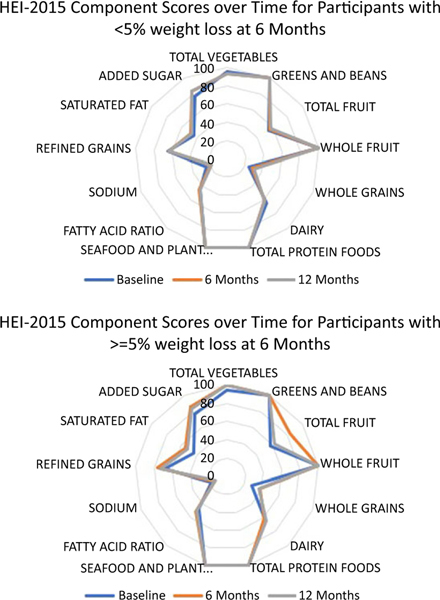
Radar Plot Depicting Component Scores of the Healthy Eating Index 2015 by 6-Month Weight Loss Status at Each Time Point. Note: Scores touching the outer ring represent the maximum score for a component (100 % of the maximum score). A perfect diet quality score of 100 would be represented by touching the outer ring for all components.

Among those at 6 months who lost ≥ 5 % of baseline weight (*n* 130) compared with those who did not (< 5 %), mean HEI-2015 scores and bootstrapped CI were greater at 6 months (67·46 (65·27, 70·12) *v*. 62·41 (60·26, 63·94), respectively) (Table 3) despite similar HEI-2015 scores at baseline (62·18 (59·47, 65·31) *v*. 62·06 (59·77, 63·67), respectively). However, by 12 months, mean HEI-2015 scores were similar by weight loss status (≥ 5% weight loss: 65·38 (62·87, 67·97) *v*. < 5 % weight loss: 62·72 (60·28, 64·43)). Component scores are presented in Fig. 2 for weight loss status at 6 months.

Results were similar when assessing weight loss status at 12 months (*n* 125). Again, at baseline, there were no differences in HEI-2015 total scores by 12-month weight loss status (≥ 5% weight loss: 62·00 (58·94, 64·12) *v*.< 5 % weight loss: 62·17 (60·26, 63·59)) (online Supplementary Table S2 and Fig. S2). Among those at 12 months who lost ≥ 5 % of baseline weight compared with those who did not (< 5 %), mean HEI-2015 scores and bootstrapped CI were greater at 6 months (68·02 (65·41, 71·23) *v*. 62·36 (60·23, 63·98), respectively) with differences slightly attenuated at 12 months (65·93 (63·40, 68·61) *v*. 62·50 (60·36, 64·41), respectively).

Among SM + FB participants (*n* 186), the median percentage of feedback messages viewed over 12 months was 50·5 %. Although CI overlapped, participants who viewed ≥ Median % of messages had slightly greater mean HEI-2015 total scores and bootstrapped CI at 6 months (68·02 (63·98, 71·15)) compared with those who viewed < Median % (63·04 (61·40, 64·61)) despite similar baseline scores (≥ Median %: 62·63 (59·73, 65·28) *v*. < Median%: 61·85 (59·66, 63·47)) (online Supplementary Table S3 and Fig. S3). By 12 months, the difference in the HEI-2015 total score was smaller (≥ Median %: 64·78 (61·05, 67·46) *v*. < Median %: 63·42 (61·52, 65·20)).

## Discussion

Little change in HEI-2015 scores was observed over 12 months in either the SM or SM + FB group. Our results are similar in magnitude to those reported in other interventions providing feedback remotely. For example, small improvements in diet Diet quality change in a mHealth intervention quality (∼2 points) have been observed at 6- and 12-month time points in the cellphone-based arm of a behavioural intervention despite specific emphasis on following the Dietary Approaches to Stop Hypertension (DASH) eating pattern, regular prompts to self-monitor and communication with ‘buddies’^(31)^. In a workplace intervention that included automated feedback, 6-, 12- and 24-month improvements in HEI-2015 total scores were similarly small (2·2, 1·8 and 1·6 points respectively)^(32)^. Additionally, in an internet-based weight loss intervention with energy and fat goals, self-monitoring and automated feedback, HEI-2015 total scores improved by approximately 4 points over 3 months^(33)^. However, as diet quality improvements have been seen to lessen over the course of an intervention^(34,35)^, additional follow-up time might have shown attenuated results.

Limited engagement with digital self-monitoring might have precluded improvement in diet quality in our study. Dietary self-monitoring declined curvilinearly over time with only about half of days being self-monitored over 12 months on average^(36)^, and the median percentage of feedback messages intervention participants viewed over the 12 months was low^(18)^. Such issues with engagement might underly the lack of differences over time as well as the lack of differences between groups since the number of diet-related feedback messages a participant receives is related to weight loss only indirectly through self-monitoring adherence^(37)^. If self-monitoring can be improved and feedback better received, results might be stronger as evidenced by the fact that we saw a signal towards greater improvement in diet quality among SM + FB participants who viewed more messages. This aligns with our previously reported results showing that percentage of feedback messages viewed is related to weight loss^(18)^.

The single session with a dietician at baseline in SMARTER may not have been sufficient for inducing diet quality change, and there were no specific goals for altering macronutrient intake, other than fat intake. Many behavioural weight loss programs such as ours, draw from the Diabetes Prevention Program (DPP). However, diet quality improvements in the DPP were small despite a high level of coach contact. This may be because only one intervention session directly addressed healthy eating independent of weight loss^(7,38)^. Indeed, it may be that improvements in diet quality were small in this study because there was no explicit focus on altering components of diet quality identified by the HEI. In a study in which sessions were specifically focussed on recommendations from the DGA, the number of sessions attended was positively associated with HEI-2005 scores^(35)^. Other reasons for the lack of improvement in diet quality in this study may be related to the fact that the intervention targeted individual-level behaviour change only. Multilevel interventions that simultaneously target individual-level and socio-ecological factors may be warranted.

Despite the lack of a significant difference between SM + FB and SM groups, the 5–6 point improvement from baseline that we observed among participants with clinically meaningful weight loss at 6 months was similar to what has been suggested as a likely meaningful difference between groups^(28)^. Our results were similar when assessing weight loss status at 12 months as 76·0 % of those who lost ≥ 5 % of baseline weight at 12 months had already lost 5 % of baseline weight by the 6-month time point.

While it is important to note a clinically meaningful cut point for improvement in diet quality has not been established and scores from different versions of the HEI (and different indices) are not directly comparable, the relationship we observed between weight loss and HEI improvement is broadly similar to that seen in other studies. In a small pilot study with a 16-week intervention period, women who achieved clinically meaningful weight loss improved HEI-2005 total scores by 8·3 points^(39)^. In both the Weight Optimization Revamping Lifestyle using the Dietary Guidelines (WORLD)^(35)^ and Daughters and Mothers Against Breast Cancer (DAMES)^(8)^ studies, which used the HEI-2005, relationships between change in diet quality and weight loss were also observed. Additionally, among rural breast cancer survivors who maintained lost weight (≥ 5 % of baseline weight) *v*. those who did not, there was a 4·6-point difference in Alternate Healthy Eating Index (AHEI) scores at 18 months^(9)^. Such accumulating evidence of a relationship between diet quality and weight loss reinforces the need for thorough consideration of diet quality in intervention design and analysis.

A scoping review of mobile-based interventions concluded there was inadequate examination of dietary behaviour change and its relationship to health outcomes^(40)^. Indeed, a systematic review of the use of the HEI in eighteen studies aimed at weight loss identified limitations in the assessment of diet^(41)^. A major strength of this study is that it is one of the few to explore this question rigorously by using multiple 24-h dietary recalls, which are less biased than food frequency questionnaires and appropriate calculation of the Healthy Eating Index. Additional strengths of the study include the randomised design, relatively large sample size and the intervention’s scalability.

### Limitations

There are some limitations to this analysis. Reporting errors cannot be ruled out as recalls were not unannounced, thus reducing one of the main advantages of using dietary recalls^(42)^. Similarly, a little less than half of participants had both a weekday and weekend dietary recall at baseline with less than a third contributing both a weekday and weekend recall at 6 and 12 months. Most participants contributed two non-consecutive recalls at baseline with only about half doing so at 6 months and 12 months. Collecting data on both weekdays/weekends and on non-consecutive days may reduce error as weekday eating has been shown to differ from weekend eating^(43)^, and consecutive days of recall tend to be correlated^(44)^. A final limitation is that our analysis included only those participants with complete data. Likely those with missing data had little to no improvement in diet quality due to their disengagement with the study. Therefore, had they been included, results might have been attenuated.

### Conclusions

In conclusion, minimal diet quality improvement was observed in this mHealth intervention among participants with overweight and obesity. When there is excessive energy intake, as with those with overweight/obesity, a focus on addressing low scores for refined grains, added sugars and/or saturated fats might be most beneficial to improving diet quality scores for two reasons. First, the general population has particularly low scores on these components. Second, as described by Krebs-Smith and colleagues, focus on these components would lead to improvements in all component scores that are density-based because refined grains, added sugars and saturated fats contribute a large number of energies^(19)^. In SMARTER, no messages addressed sodium and few addressed grains (refined or whole) or saturated fat specifically. Therefore, more focussed feedback messages might help induce changes in diet quality.

Personalisation of features besides feedback, such as delivery or timing,^(45)^ increasing self-efficacy and culturally tailoring nutritional feedback have also been suggested as ways of increasing the benefits of remote, personalised interventions^(46)^. However, because diet quality improvement in weight loss trials has not been extensively explored, components of interventions necessary for producing diet quality improvement remain unclear. As such, weight loss researchers may consider assessing improvements in diet quality such that clarity can be achieved. Widening focus when designing interventions to include improvement not only for weight and other cardiometabolic outcomes, but also for health behaviours such as diet quality, may benefit participants having difficulty achieving or maintaining weight loss.

## Supplementary Material

1

## Figures and Tables

**Table 1.** Baseline characteristics of SMARTER study participants by dietary data missingness status

Characteristic	Total sample *n* 502		Complete dietary data *n* 356		Missing dietary data† *n* 146		*P*-value

	*n*/Median	%/p25, p75	*n*/Median	%/p25, p75	*n*/Median	%/p25, p75	
Demographic characteristics							
Age, years	45.5	32.0, 57.0	51.0	36 0, 60 0	35.0	29 0, 46 0	< 0 0001*
Sex							
Male	103	20.5	75	21.1	28	19.2	0.63
Female	399	79.5	281	78.9	118	80.8	
Race and ethnicity							
Asian	14	2.8	8	2.3	6	4.1	0.01*
Black	48	9.6	32	9.0	16	11.0	
Multiracial§	26	5.2	12	3.4	14	9.6	
White	414	82.5	304	85.4	110	75.3	
Education, years							
Mean	16.4		16.5		16.4		0.72
SD	2.8		2.8		2.9		
Employment							
Full-time/part-time	412	82.1	291	81 7	121	82.9	0.76
Unemployed‡	90	17.9	65	18.3	25	17.1	
Annual household income							
< $60 000	143	28.5	97	30.0	46	34.6	0.34
≥ $60 000	313	62.4	226	70.0	87	65.4	
Clinical characteristics (self-reported)							
High blood pressure	106	21.1	87	24.4	19	13.0	< 0.01*
High cholesterol	88	17.5	74	20.8	14	9.6	< 0.01*
High TAG	56	11.2	44	12.4	12	8.2	0.18
Smoking status							
Never smoker	385	76.7	273	76.7	112	76.7	0.48
Current smoker	14	2.8	8	2.3	6	4.1	
Former smoker	103	20.5	75	21.1	28	19.2	
Anthropometric measures							
BMI, kg/m^2^	33.3	30 7, 36 6	33.1	30 3, 36 2	33.8	31.0, 37.5	0.03*
	Mean	SD	Mean	SD	Mean	SD	
Average waist circumference, cm							
Men	113.1	12.2	112.5	11.9	114.7	13.0	0.43
Women	105.3	11.1	105.5	10.5	104.9	12.4	0.62
Average systolic blood pressure, mmHg	118.2	15.4	119.1	15.1	116.0	16.0	0.04*
Average diastolic blood pressure, mmHg	77.0	10.8	76.9	10.8	77.0	10.9	0.97

Notes: BMI, Body mass index; TAG, triglycerdies. For categorical variables, *P*-values were obtained using the chi-square test of independence. For age, BMI and body fat percentage the Wilcoxon rank-sum test were used to assess differences by missingness category because of non-normality identified during visualisation of histograms and q–q plots. Two-sided *P*-values are presented. For all other continuous variables, two sample *t* tests were used to assess differences by missingness category as plots suggested normality. For all continuous variables except female waist circumference, the pooled variance method was used as variances were equal. Separate-variance *t* tests with the Satterthwaite method approximation of degrees of freedom were used for female waist circumference.

* Statistically significant at the *P* < 0·05 level.

† Dietary data were missing at 6 and/or 12 months if there were 0 recalls collected at that timepoint.

‡ The Unemployed category includes participants who were unemployed, retired or disabled.

§ Indicates participants who self-reported ‘yes’ to a question, ‘Are you of more than one racial/ethnic background?’.

**Table 2.** HEI-2015 total and component scores by treatment group and time point

		Baseline		6 months		12 months	
	Study arm	Mean	95 % CI	Mean	95 % CI	Mean	95 % CI
Total HEI-2015 score	SM	61·02	58·72, 62·81	63·19	61·22, 64·97	63·56	60·81, 65·42
	SM + FB	63·11	60·41, 65·24	65·42	63·30, 67·20	63·94	61·40, 66·29
Adequacy components							
Total vegetables	SM	4·76	4·29, 5·00	5·00	4·57, 5·00	5·00	4·79, 5·00
	SM + FB	4·71	4·33, 5·00	5·00	4·81, 5·00	4·78	4·37, 5·00
Greens and beans	SM	5·00	4·85, 5·00	5·00	5·00, 5·00	5·00	5·00, 5·00
	SM + FB	5·00	5·00, 5·00	5·00	5·00, 5·00	5·00	5·00, 5·00
Total fruit	SM	2·80	2·39, 3·22	3·34	2·89, 3·87	2·97	2·48, 3·39
	SM + FB	2·81	2·35, 3·30	3·17	2·76, 3·81	3·01	2·57, 3·46
Whole fruit	SM	5·00	4·28, 5·00	5·00	5·00, 5·00	5·00	4·29, 5·00
	SM + FB	5·00	4·31, 5·00	5·00	4·88, 5·00	5·00	4·68, 5·00
Whole grains	SM	2·39	2·02, 2·82	2·80	2·37, 3·32	3·12	2·61, 3·53
	SM + FB	2·93	2·34, 3·38	3·57	2·99, 4·04	3·55	2·93, 4·23
Dairy	SM	6·22	5·72, 6·73	6·02	5·43, 6·68	6·58	5·94, 7·20
	SM + FB	6·57	6·09, 7·04	6·08	5·49, 6·63	5·89	5·45, 6·42
Total protein foods	SM	5·00	5·00, 5·00	5·00	5·00, 5·00	5·00	5·00, 5·00
	SM + FB	5·00	5·00, 5·00	5·00	5·00, 5·00	5·00	5·00, 5·00
Seafood and plant protein	SM	5·00	4·97, 5·00	5·00	4·94, 5·00	5·00	5·00, 5·00
	SM + FB	5·00	5·00, 5·00	5·00	5·00, 5·00	5·00	5·00, 5·00
Moderation components							
Fatty acid ratio	SM	4·26	3·61, 4·92	4·50	3·92, 5·10	4·20	3·63, 4·95
	SM + FB	4·76	4·11, 5·40	4·97	4·32, 5·66	5·07	4·44, 5·63
Sodium	SM	2·22	1·56, 2·91	1·94	1·24, 2·69	1·85	1·06, 2·57
	SM + FB	2·21	1·52, 2·81	1·59	0·82, 2·31	1·46	0·72, 2·08
Refined grains	SM	6·74	5·97, 7·38	6·60	5·92, 7·21	7·07	6·48, 7·81
	SM + FB	6·60	5·87, 7·31	7·12	6·36, 7·83	6·48	5·78, 7·04
Saturated fat	SM	4·50	3·77, 5·07	4·58	3·88, 5·26	4·63	4·04, 5·28
	SM + FB	4·39	3·80, 5·02	5·53	5·00, 6·12	5·17	4·51, 5·85
Added sugar	SM	7·14	6·50, 7·68	8·40	7·91, 8·86	8·14	7·57, 8·73
	SM + FB	8·15	7·76, 8·62	8·39	8·01, 8·77	8·54	8·12, 8·91

HEI-2015, Healthy Eating Index-2015, SM, Self-monitoring group, SM + FB, self-monitoring + feedback group.

95 % CI are based on bootstrapped resamples.

**Table 3.** HEI-2015 scores at each time point by weight loss status at 6 months

		Baseline		6 months		12 months	
	Weight loss status at 6 months	Mean	95 % CI	Mean	95 % CI	Mean	95 % CI
Total HEI-2015 score	< 5%	62·06	59·77, 63·67	62·41	60·26, 63·94	62·72	60·28, 64·43
	≥ 5%	62·18	59·47, 65·31	67·46	65·27, 70·12	65·38	62·87, 67·97
Adequacy components							
Total vegetables	< 5%	4·66	4·30, 5·00	4·68	4·35, 5·00	4·74	4·36, 5·00
	≥ 5%	4·73	4·28, 5·00	5·00	5·00, 5·00	4·73	4·28, 5·00
Greens and beans	< 5%	5·00	5·00, 5·00	5·00	5·00, 5·00	5·00	5·00, 5·00
	≥ 5%	5·00	5·00, 5·00	5·00	5·00, 5·00	5·00	5·00, 5·00
Total fruit	< 5%	2·76	2·41, 3·16	2·80	2·48, 3·11	2·90	2·51, 3·25
	≥ 5%	2·89	2·41, 3·43	4·15	3·54, 4·98	3·16	2·61, 3·60
Whole fruit	< 5%	5·00	4·35, 5·00	5·00	4·29, 5·00	5·00	4·38, 5·00
	≥ 5%	5·00	4·39, 5·00	5·00	5·00, 5·00	5·00	4·80, 5·00
Whole grains	< 5%	2·56	2·19, 2·91	2·85	2·43, 3·26	3·16	2·72, 3·63
	≥ 5%	2·85	2·26, 3·49	3·87	3·17, 4·67	3·69	3·04, 4·56
Dairy	< 5%	6·45	6·02, 6·92	6·03	5·44, 6·52	6·11	5·64, 6·61
	≥ 5%	6·30	5·67, 7·08	6·11	5·56, 6·85	6·39	5·83, 6·99
Total protein foods	< 5%	5·00	5·00, 5·00	5·00	5·00, 5·00	5·00	5·00, 5·00
	≥ 5%	5·00	5·00, 5·00	5·00	5·00, 5·00	5·00	5·00, 5·00
Seafood and plant protein	< 5%	5·00	5·00, 5·00	5·00	5·00, 5·00	5·00	5·00, 5·00
	≥ 5%	5·00	5·00, 5·00	5·00	5·00, 5·00	5·00	5·00, 5·00
Fatty acid ratio	< 5%	4·44	3·79, 5·14	4·56	4·05, 5·14	4·52	4·07, 5·09
	≥ 5%	4·66	3·94, 5·61	5·10	4·31, 5·94	4·89	4·19, 5·73
Moderation components							
Sodium	< 5%	2·47	1·90, 3·12	1·92	1·23, 2·53	1·82	1·14, 2·36
	≥ 5%	1·78	1·02, 2·76	1·45	0·48, 2·33	1·33	0·51, 2·30
Refined grains	< 5%	6·55	5·88, 7·16	6·45	5·89, 7·01	6·44	5·83, 6·98
	≥ 5%	6·86	6·06, 7·71	7·69	6·93, 8·50	7·32	6·55, 8·16
Saturated fat	< 5%	4·43	3·81, 5·00	4·84	4·29, 5·38	4·76	4·25, 5·29
	≥ 5%	4·46	3·66, 5·21	5·50	4·72, 6·30	5·19	4·45, 5·87
Added sugar	< 5%	7·67	7·22, 8·20	8·29	7·87, 8·64	8·32	7·87, 8·72
	≥ 5%	7·65	7·04, 8·22	8·59	8·13, 8·96	8·40	7·88, 8·89

HEI-2015, Healthy Eating Index-2015.

95 % CI are based on bootstrapped resamples.

## Abbreviations:

HEI-2015: Healthy Eating Index 2015
