# Supplementary material for: Effect of an mHealth weight loss intervention on Healthy Eating Index diet quality: the SMARTER randomised controlled trial
Source: Br J Nutr. Author manuscript; Available in PMC 2024 Jun 14. (PMC10632722; doi:10.1017/S0007114523001137)
Supplement: 1 [file NIHMS1896472-supplement-1.docx]

| **Supplemental Table S1: Example Dietary Feedback Messages Addressing Components of the Healthy Eating Index-2015** | |
| --- | --- |
| **Adequacy Components** | **Example Message** |
| Total Fruits | Eating enough for breakfast plays a big role in energizing your day! If you already ate breakfast, you could **choose some fruit for a snack.** |
| Whole Fruits | Do you like juice? Juice is a concentrated source of sugar. There are at least six oranges in a one cup of orange juice. **Choose to eat the whole fruit instead.** It will offer more filling fiber and nutrients found in the pulp and skin. |
| Total Vegetables | Choose foods lower in fat tonight to stay closer to your fat gram goal! **Steamed veggies or a big salad could be good options** to incorporate. |
| Greens and Beans | Fat intake is looking good! Take a moment to plan low calorie foods for this afternoon. A lean meat or **beans with steamed vegetables are good options.** |
| Whole Grains | If you are still under your calorie goal, you have some room for healthy fats! Nut butter on a **whole grain cracker or bread might be a good option.** |
| Dairy | Excellent job recording food intake. **Sugars look high. Some could have come from milk, yogurt**, sweets, and beverages. Can you identify where yours came from today? |
| Total Protein Foods | Way to self-monitor throughout the day. Sugars are higher today. Choose low-sugar, **high-protein foods such as eggs, meat, or hummus** with vegetables to snack on this evening. |
| Seafood and Plant Proteins | You are doing amazing recording, and your calories are on track. You could add some fat grams- **if you like fish, seafood can be a great way to add healthy fats to your diet**. If you don’t like seafood, think about **plant-based fats such as those found in nuts and avocados.** |
| Fatty Acids | Calories are on target! Fat grams are higher than your goal. Make swaps by **selecting lean protein sources where you may have had fatty protein sources**, or vegetables where you may have had cheese |
| **Moderation Components** | **Example Message** |
| Refined Grains | You are a self-monitoring pro! Take a peek at your log, calories are above your goal range and fat grams are low. **Swapping something like pasta** for a healthy salad with oil-based dressing could balance your intake and help you to meet both goals! |
| Sodium | No messages |
| Added Sugars | Are you drinking **sweetened beverages**? Did you know that one 16 fluid ounce bottle of soda can contain 13 teaspoons of sugar, whoa! |
| Saturated Fats | Calories are on target, while fat grams are a bit low for this time of day. If you are **avoiding saturated fat (e.g., butter, bacon)** to keep your calories in check, remember that healthy fats (fish, nuts, avocados) can also satisfy you and fuel your body. If you choose the right portion size, your calorie intake should be fine. |


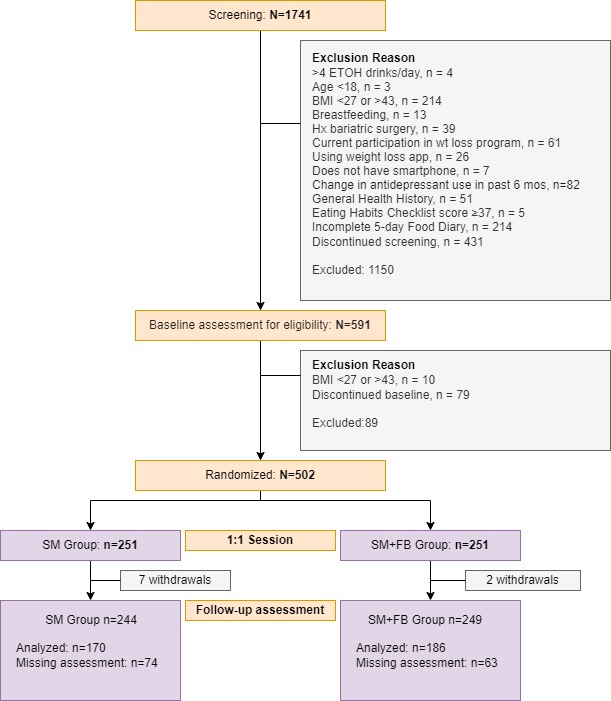


**Supplemental Figure S1: CONSORT (Consolidated Standards of Reporting Trials) flow diagram for SMARTER Study Participants Included in a Secondary Analysis of Diet Quality Outcomes.**

| **Supplemental Table S2: HEI-2015 Scores at Each Time Point by Weight Loss Status at 12 Months** | | | | |
| --- | --- | --- | --- | --- |
|  | **Weight Loss Status**  **at 12 Months** | **Baseline**  **Mean [95% CI]** | **6 Months**  **Mean [95% CI]** | **12 Months**  **Mean [95% CI]** |
| TOTAL HEI-2015 SCORE | <5% | 62.17  (60.26, 63.59) | 62.36  (60.23, 63.98) | 62.50  (60.36, 64.41) |
|  | ≥5% | 62.00  (58.94, 64.12) | 68.02  (65.41, 71.23) | 65.93  (63.40, 68.61) |
| **Adequacy Components** | | | | |
| TOTAL VEGETABLES | <5% | 4.77  (4.43, 5.00) | 4.81  (4.47, 5.00) | 4.69  (4.31, 5.00) |
|  | ≥5% | 4.67  (4.13, 5.00) | 5.00  (5.00, 5.00) | 5.00  (4.99, 5.00) |
| GREENS AND BEANS | <5% | 5.00  (5.00, 5.00) | 5.00  (5.00, 5.00) | 5.00  (5.00, 5.00) |
|  | ≥5% | 5.00  (5.00, 5.00) | 5.00  (5.00, 5.00) | 5.00  (5.00, 5.00) |
| TOTAL FRUIT | <5% | 2.82  (2.40, 3.21) | 2.86  (2.49, 3.25) | 2.89  (2.58, 3.23) |
|  | ≥5% | 2.77  (2.33, 3.30) | 4.08  (3.41, 4.75) | 3.19  (2.59, 3.78) |
| WHOLE FRUIT | <5% | 5.00  (4.36, 5.00) | 5.00  (4.35, 5.00) | 5.00  (4.41, 5.00) |
|  | ≥5% | 5.00  (4.26, 5.00) | 5.00  (5.00, 5.00) | 5.00  (4.70, 5.00) |
| WHOLE GRAINS | <5% | 2.51  (2.18, 2.89) | 2.98  (2.50, 3.42) | 3.18  (2.72, 3.67) |
|  | ≥5% | 2.96  (2.45, 3.49) | 3.64  (3.08, 4.30) | 3.67  (2.90, 4.50) |
| DAIRY | <5% | 6.30  (5.90, 6.69) | 5.79  (5.31, 6.32) | 6.22  (5.70, 6.68) |
|  | ≥5% | 6.58  (5.93, 7.28) | 6.60  (5.95, 7.25) | 6.19  (5.65, 6.71) |
| TOTAL PROTEIN FOODS | <5% | 5.00  (5.00, 5.00) | 5.00  (5.00, 5.00) | 5.00  (5.00, 5.00) |
|  | ≥5% | 5.00  (5.00, 5.00) | 5.00  (5.00, 5.00) | 5.00  (5.00, 5.00) |
| SEAFOOD AND PLANT PROTEIN | <5% | 5.00  (5.00, 5.00) | 5.00  (5.00, 5.00) | 5.00  (5.00, 5.00) |
|  | ≥5% | 5.00  (5.00, 5.00) | 5.00  (5.00, 5.00) | 5.00  (5.00, 5.00) |
| FATTY ACID RATIO | <5% | 4.57  (3.98, 5.21) | 4.62  (4.16, 5.14) | 4.41  (3.95, 4.99) |
|  | ≥5% | 4.42  (3.63, 5.11) | 4.99  (4.01, 5.99) | 5.13  (4.37, 5.90) |
| **Moderation Components** | | | | |
| SODIUM | <5% | 2.53  (1.92, 3.00) | 1.86  (1.26, 2.50) | 1.95  (1.34, 2.55) |
|  | ≥5% | 1.64  (0.85, 2.50) | 1.56  (0.60, 2.47) | 1.06  (0.26, 1.89) |
| REFINED GRAINS | <5% | 6.52  (5.93, 7.06) | 6.41  (5.95, 7.06) | 6.40  (5.79, 7.01) |
|  | ≥5% | 6.93  (5.98, 7.59) | 7.81  (6.76, 8.74) | 7.45  (6.74, 8.25) |
| SATURATED FAT | <5% | 4.49  (3.99, 5.02) | 4.75  (4.23, 5.26) | 4.73  (4.20, 5.31) |
|  | ≥5% | 4.35  (3.46, 5.25) | 5.73  (4.76, 6.50) | 5.28  (4.44, 5.99) |
| ADDED SUGAR | <5% | 7.65  (7.15, 8.14) | 8.28  (7.86, 8.63) | 8.03  (7.64, 8.45) |
|  | ≥5% | 7.67  (7.11, 8.27) | 8.62  (8.18, 9.04) | 8.96  (8.49, 9.46) |
| CI, Confidence interval, HEI-2015, Healthy Eating Index-2015  Note: 95% CI are based on bootstrapped resamples | | | | |

**Supplementary Figure S2: Radar Plot Depicting Component Scores of the Healthy Eating Index 2015 by 12-Month Weight Loss Status at Each Time Point**


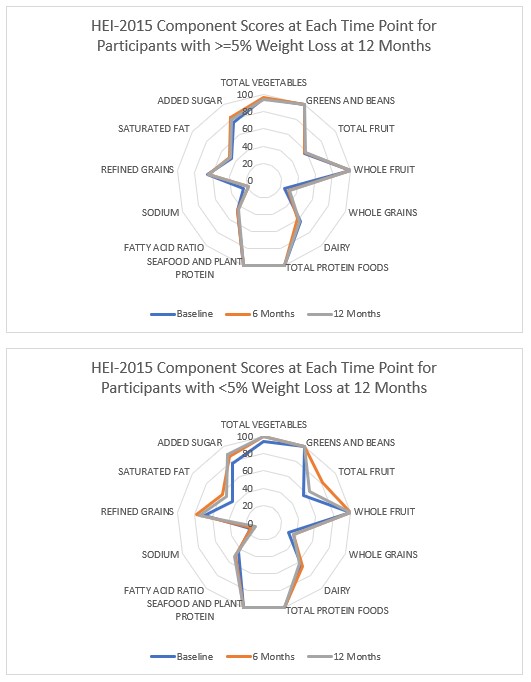


Note: Scores touching the outer ring represent the maximum score for a component (100% of the maximum score). A perfect diet quality score of 100 would be represented by touching the outer ring for all components.

| **Supplemental Table S3: HEI-2015 Scores at Each Time Point by Percent of Feedback Messages Viewed** | | | | |
| --- | --- | --- | --- | --- |
|  | **% of Feedback Messages Viewed** | **Baseline**  **Mean [95% CI]** | **6 Months**  **Mean [95% CI]** | **12 Months**  **Mean [95% CI]** |
| TOTAL HEI-2015 SCORE | <Median % | 61.85  (59.66, 63.47) | 63.04  (61.40, 64.61) | 63.42  (61.52, 65.20) |
|  | ≥Median % | 62.63  (59.73, 65.28) | 68.02  (63.98, 71.15) | 64.78  (61.05, 67.46) |
| **Adequacy Components** | | | | |
| TOTAL VEGETABLES | <Median % | 4.76  (4.42, 5.00) | 5.00  (4.70, 5.00) | 5.00  (4.83, 5.00) |
|  | ≥Median % | 4.67  (4.10, 5.00) | 5.00  (4.89, 5.00) | 4.73  (4.15, 5.00) |
| GREENS AND BEANS | <Median % | 5.00  (5.00, 5.00) | 5.00  (5.00, 5.00) | 5.00  (5.00, 5.00) |
|  | ≥Median % | 5.00  (5.00, 5.00) | 5.00  (5.00, 5.00) | 5.00  (5.00, 5.00) |
| TOTAL FRUIT | <Median % | 2.68  (2.36, 3.02) | 3.17  (2.74, 3.61) | 2.93  (2.58, 3.24) |
|  | ≥Median % | 3.14  (2.44, 3.96) | 3.49  (2.65, 4.32) | 3.18  (2.59, 3.74) |
| WHOLE FRUIT | <Median % | 4.93  (4.31, 5.00) | 5.00  (4.90, 5.00) | 5.00  (4.57, 5.00) |
|  | ≥Median % | 5.00  (4.37, 5.00) | 5.00  (4.72, 5.00) | 5.00  (4.65, 5.00) |
| WHOLE GRAINS | <Median % | 2.63  (2.33, 2.96) | 3.09  (2.65, 3.60) | 3.25  (2.86, 3.65) |
|  | ≥Median % | 2.78  (2.16, 3.39) | 3.51  (2.72, 4.18) | 3.61  (2.80, 4.29) |
| DAIRY | <Median % | 6.24  (5.87, 6.70) | 6.02  (5.60, 6.50) | 6.36  (5.96, 6.89) |
|  | ≥Median % | 6.82  (6.10, 7.58) | 6.16  (5.50, 6.85) | 5.82  (5.17, 6.43) |
| TOTAL PROTEIN FOODS | <Median % | 5.00  (5.00, 5.00) | 5.00  (5.00, 5.00) | 5.00  (5.00, 5.00) |
|  | ≥Median % | 5.00  (5.00, 5.00) | 5.00  (5.00, 5.00) | 5.00  (5.00, 5.00) |
| SEAFOOD AND PLANT PROTEIN | <Median % | 5.00  (5.00, 5.00) | 5.00  (5.00, 5.00) | 5.00  (5.00, 5.00) |
|  | ≥Median % | 5.00  (5.00, 5.00) | 5.00  (5.00, 5.00) | 5.00  (5.00, 5.00) |
| FATTY ACID RATIO | <Median % | 4.51  (3.98, 5.00) | 4.45  (3.97, 5.02) | 4.46  (3.89, 5.04) |
|  | ≥Median % | 4.54  (3.69, 5.72) | 5.60  (4.57, 6.63) | 5.15  (4.29, 5.99) |
| **Moderation Components** | | | | |
| SODIUM | <Median % | 2.45  (1.92, 3.00) | 1.65  (0.99, 2.25) | 1.55  (0.88, 2.17) |
|  | ≥Median % | 1.58  (0.70, 2.57) | 2.08  (1.03, 2.96) | 1.89  (0.90, 2.71) |
| REFINED GRAINS | <Median % | 6.68  (6.06, 7.11) | 6.62  (6.06, 7.27) | 6.77  (6.29, 7.35) |
|  | ≥Median % | 6.64  (5.72, 7.54) | 7.56  (6.71, 8.49) | 6.72  (5.84, 7.52) |
| SATURATED FAT | <Median % | 4.49  (3.97, 4.97) | 4.70  (4.22, 5.31) | 4.82  (4.26, 5.35) |
|  | ≥Median % | 4.31  (3.29, 5.20) | 6.09  (5.41, 6.91) | 5.17  (4.30, 5.92) |
| ADDED SUGAR | <Median % | 7.48  (7.02, 7.92) | 8.35  (7.98, 8.71) | 8.29  (7.87, 8.66) |
|  | ≥Median % | 8.15  (7.51, 8.76) | 8.53  (8.03, 9.06) | 8.50  (7.92, 9.04) |
| CI, Confidence interval, HEI-2015, Healthy Eating Index-2015  Note: 95% CI are based on bootstrapped resamples. The median percent of feedback messages viewed was 50.5%. | | | | |

**Supplementary Figure S3: Radar Plot Depicting Component Scores of the Healthy Eating Index 2015 by Percent of Feedback Messages Viewed over 12 Months**


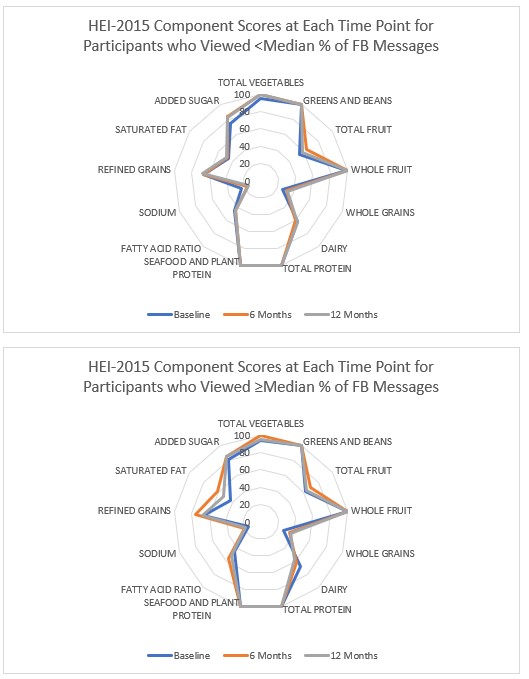


Note: Scores touching the outer ring represent the maximum score for a component (100% of the maximum score). A perfect diet quality score of 100 would be represented by touching the outer ring for all components. The median percentage of feedback messages viewed over 12 months was 50.5%.
